# Reply to the Letter

**DOI:** 10.1007/s00062-015-0466-z

**Published:** 2015-09-16

**Authors:** H. Kim, H.S. Hong

**Affiliations:** Department of Radiology, Soonchunhyang University Bucheon Hospital, 170 Jomaru-ro, Wonmi-gu, Bucheon-si, Gyeonggi-do, 420-767 Bucheon, Republic of Korea

We agree that perfluorocarbon liquid (PFCL) should be used only temporarily in an intraoperative context, and should be removed before the operation is concluded. We did not use PFCL when operating on our patient. Rather, the patient was given an injection of 5500cST silicone oil, as shown in Fig. 1. The patient did not have any predisposing anatomical factor. The optic disc was not deeply cupped and no congenital optic pits were evident on funduscopic examination.

The intraocular pressure normalised 18 days after intraocular tamponade. Two months later, we found that the intraocular pressure was poorly controlled; this situation persisted for a further 8 months. Therefore, we scheduled the patient for a second surgery.

